# Real-time inextensible surgical thread simulation

**DOI:** 10.1007/s11548-018-1739-1

**Published:** 2018-03-27

**Authors:** Lang Xu, Qian Liu

**Affiliations:** 1 Britton Chance Center for Biomedical Photonics, School of Engineering Sciences, Wuhan National Laboratory for Optoelectronics-Huazhong University of Science and Technology, Wuhan, 430074 China; 2 Key Laboratory for Biomedical Photonics, Huazhong University of Science and Technology, Ministry of Education, 1037 Luoyu Road, Wuhan, Hubei 430074 China

**Keywords:** Cosserat rod, Surgical thread, Virtual surgery, Position-based dynamics

## Abstract

**Purpose:**

This paper discusses a real-time simulation method of inextensible surgical thread based on the Cosserat rod theory using position-based dynamics (PBD). The method realizes stable twining and knotting of surgical thread while including inextensibility, bending, twisting and coupling effects.

**Methods:**

The Cosserat rod theory is used to model the nonlinear elastic behavior of surgical thread. The surgical thread model is solved with PBD to achieve a real-time, extremely stable simulation. Due to the one-dimensional linear structure of surgical thread, the direct solution of the distance constraint based on tridiagonal matrix algorithm is used to enhance stretching resistance in every constraint projection iteration. In addition, continuous collision detection and collision response guarantee a large time step and high performance. Furthermore, friction is integrated into the constraint projection process to stabilize the twining of multiple threads and complex contact situations.

**Results:**

Through comparisons with existing methods, the surgical thread maintains constant length under large deformation after applying the direct distance constraint in our method. The twining and knotting of multiple threads correspond to stable solutions to contact and friction forces. A surgical suture scene is also modeled to demonstrate the practicality and simplicity of our method.

**Conclusions:**

Our method achieves stable and fast simulation of inextensible surgical thread. Benefiting from the unified particle framework, the rigid body, elastic rod, and soft body can be simultaneously simulated. The method is appropriate for applications in virtual surgery that require multiple dynamic bodies.

## Introduction

Surgical thread is a valuable material used during surgery that helps reduce tissue damage and accelerate wound healing. Existing suture training methods involve plastic materials or animal tissues as an alternative to human soft tissue. Virtual surgery systems serve as practical training aides for clinical operations, helping novices familiarize themselves with medical instruments and entire operation procedures by repeatedly simulating surgical maneuvers in a safe environment [1]. Therefore, an appropriate surgical thread simulation is necessary for improving virtual surgery systems. Training protocols that include suture steps in the procedure provide a more complete learning experience that enhances trainee immersion.

Surgical thread is represented as an elastic rod because it has an axial length that is much larger than the other two dimensions. An elastic rod exhibits typical nonlinear characteristics when deformed and can exhibit large bending and twisting strain with nearly constant length. The conventional elastic rod model is described by the Cosserat theory. A Cosserat rod is a physical model that describes an elastic rod utilizing the material frame and directional curve [2]. The primary goal of the real-time simulation of surgical thread is to quickly and stably represent the typical characteristics of real surgical thread, such as the coupling effects of bending and twisting and inextensible behavior.

The computer graphics community has devoted significant efforts toward building a real-time elastic rod; hair and rope simulations have been developed, and several applications in the field of game have modeled real-time elastic rods [3–5]. For example, the mass spring model (MSM) and the finite element model (FEM) have been optimized to construct hair and rope physical models [6, 7]. The MSM model is computationally fast but requires more complex topologies to support nonlinear behavior, which includes the bending and twisting of elastic rods. The FEM model, based on the Euler beam, has high accuracy but requires intensive calculations and a relatively small time step [8]. Therefore, Spillmann and Teschner introduced the simulation of Cosserat rod elements based on strain energy using the FEM model [2]. Additionally, Bergou et al. [9] manipulated the centerline of the elastic rod to yield a discrete format of the bend and twist energy equations. These methods have a high accuracy and can represent complex nonlinear deformation behaviors, although the numerical steps are time consuming. Some experts have applied these methods to real-time surgical thread simulations. Wang et al. [10] used the Kirchhoff rod to describe surgical thread with an inextensibility constraint, but this method was unable to achieve an extremely stable simulation to accommodate a large time step. By considering the high stability of position-based dynamics (PBD), a large time step can be achieved [11]. Previously, Kubiak et al. [12] introduced a method based on PBD to simulate surgical thread, which allows fast and stable simulations, but it cannot describe the coupling effects of bending and twisting due to a weak physical foundation. Following this method, ghost points were introduced by Umetani el al. to represent the material frame as particles [13]. However, the bending and twisting constraint gradient directions have complicated formats, and computing them is time consuming. After quaternion is introduced into PBD, the constraint is expanded from a scalar-valued function to a vector-valued function [14]. In a recent study, the material frame was parametrized as a quaternion. Thus, the format of the constraint gradient becomes concise, and the complexity is reduced during the constraint projection [15]. However, due to the fixed and limited iteration count used in the Gauss–Seidel method, its convergence cannot be guaranteed, causing the constant length requirement for the elastic rod to not always be fulfilled.

The scale of threads for surgery simulation is considerably lower than that for real-time graphics applications. However, surgical thread simulation has more requirements for expression precision related to the thread to thread interactions, thread to soft body and rigid body interactions, which involves collision detection and computing friction for multiple objects. Furthermore, in the representation of mechanical properties, bending and twisting coupling effects and inextensible behavior should be considered. These factors make the surgical thread simulations unique with distinct complexity. In addition to represent the surgical thread, the inextensible Cosserat rod can also be used for simulating other medical instruments such as interventional guidewire and gastric endoscope [16, 17]. Based on PBD, surgical thread simulation can be easily integrated into existing soft tissue deform simulation, representing a highly practical approach for medical development applications.

The method proposed in this paper simulates surgical thread based on the Cosserat rod theory using PBD. The method is fast and extremely stable, which are benefits of PBD. Additionally, the simulated surgical thread exhibits coupled bending and twisting effects. The tridiagonal matrix algorithm is used to solve the direct solution of the distance constraint, and the constraint of inextensible behavior can be realized in a single iteration. The three key innovations in this paper are as follows:

- The use of PBD and continuous collision detection to achieve a large time step stable simulation of surgical thread with high computational performance;
- The use of the one-dimensional linear geometric feature of surgical thread to obtain strongly constrained inextensibility by directly solving the problem based on the tridiagonal matrix algorithm;
- The use of the unified particle framework, including an elastic rod, soft body and rigid body, to simulate surgical suturing in a real-time application for fast, concise implementation.

This paper is organized into three parts. First, we introduce the method applying the Cosserat rod theory to PBD, explain how to fulfill the distance constraint using the tridiagonal matrix algorithm, and present continuous collision detection and responses, supporting a large time step. Additionally, the visual rendering method and simulation environment constructing are clarified. Second, the results section compares simulations effect of three different types of elastic rods using PBD with and without the inextensible effect, presents simulations of tying a squared knot and of multiple threads intertwining with a rigid body, and shows the construction of a surgical suture scene. Third, the final section discusses the advantages and disadvantages of our method and considers its possible future applications.

## Methods

### Vector-value constraint-based PBD

PBD has been extensively used in applications ranging from soft body simulation to rigid body and fluid simulations [18]. Overall, PBD still is a constraint-based optimization problem. However, the basic independent variable is expressed as two forms: a position vector and an orientation quaternion. The dependent variable is a vector value. Here, we clarify the unified particle framework based on PBD; more detailed descriptions of the improved process can be found in previous works [11, 13–15, 18].

The body simulated is constructed as particles represented by positions and orientations. Position is defined by the parameterized value ${{\varvec{x}}}=\left[ {{{\varvec{x}}}_1 ,{{\varvec{x}}}_2 ,\ldots ,{{\varvec{x}}}_{{j}} } \right]$ and orientation by ${{\varvec{q}}}=\left[ {{{\varvec{q}}}_1 ,{{\varvec{q}}}_2 ,\ldots ,{{\varvec{q}}}_{{k}} } \right]$. These two forms are unified as $\mathbf{p}=\left[ {{{\varvec{p}}}_1 ,{{\varvec{p}}}_2 ,\ldots ,{{\varvec{p}}}_{{n}} } \right]$, and the constraints are unified as ${{\varvec{c}}}=\left[ {{{\varvec{c}}}_1 ,{{\varvec{c}}}_2 ,\ldots ,{{\varvec{c}}}_{{m}} } \right]$. In the iteration process, the problem to be solved is corrected for position and orientation to yield:

1 $$\begin{aligned} {{\varvec{C}}}\left( {{{\varvec{p}}}+\Delta {{\varvec{p}}}} \right) \approx {{\varvec{C}}}\left( {{\varvec{p}}} \right) +\nabla {{\varvec{C}}}\left( {{\varvec{p}}} \right) \Delta {{\varvec{p}}}=\mathbf{0}. \end{aligned}$$

As the number of unknown quantities is larger than the number of equations in Eq. (1), the system state of the simulated body is underdetermined. Considering the conservation of linear and angular momentum, the correction direction of the independent variable is limited to be parallel to the direction of the gradient. Compared to the scalar constraint, the gradient of the vector-value constraint is replaced as m rows of a matrix instead of a single-row vector. Then, we introduce the Lagrange multiplier and the inverse of the mass and moment of inertia $\mathbf{W}=\hbox {diag}\left[ {{m}_1^{-1} ,{m}_2^{-1} ,\ldots ,{m}_j^{-1} ,\mathbf{I}_1^{-1} ,\mathbf{I}_2^{-1} ,\ldots ,\mathbf{I}_k^{-1} } \right]$ to weight the position and orientation:

2 $$\begin{aligned} \Delta {{\varvec{p}}}=\mathbf{W}\nabla {{\varvec{C}}}\left( {{\varvec{p}}} \right) ^{\mathrm{T}}{\varvec{\lambda }}. \end{aligned}$$

Substituting Eq. (2) into Eq. (1):

3 $$\begin{aligned} {{\varvec{C}}}\left( {{\varvec{p}}} \right) +\nabla {{\varvec{C}}}\left( {{\varvec{p}}} \right) \mathbf{W}\nabla {{\varvec{C}}}\left( {{\varvec{p}}} \right) ^{\mathrm{T}}{\varvec{\lambda }} =\mathbf{0}. \end{aligned}$$

Then, solving for ${\varvec{\lambda }}$:

4 $$\begin{aligned} {\varvec{\lambda }} =-\left( {\nabla {{\varvec{C}}}\left( {{\varvec{p}}} \right) \mathbf{W}\nabla {{\varvec{C}}}\left( {{\varvec{p}}} \right) ^{\mathrm{T}}} \right) ^{-1}{{\varvec{C}}} \left( {{\varvec{p}}} \right) \!. \end{aligned}$$

Substituting ${\varvec{\lambda }}$ into Eq. (2) to obtain the correction $\Delta {{\varvec{p}}}$:

5 $$\begin{aligned} {{\Delta }} {\varvec{p}}=-\mathbf{W}\nabla {{\varvec{C}}}\left( {{\varvec{p}}} \right) ^{{\mathrm{T}}}\left( {\nabla {{\varvec{C}}}\left( {{\varvec{p}}} \right) \mathbf{W}\nabla {{\varvec{C}}}\left( {{\varvec{p}}} \right) ^{{\mathrm{T}}}} \right) ^{-1}{{\varvec{C}}}\left( {{\varvec{p}}} \right) \!. \end{aligned}$$

These equations are used to update the positions and orientations. In most situations, the constraint functions are not linear forms of positions or orientations. Therefore, it is often impossible to directly obtain the system state that satisfies all the constraint functions. Using the Gauss–Seidel method, the system state can be gradually corrected to satisfy the constraint functions in each iteration. Although the fixed iteration count cannot guarantee that the constraint functions are completely satisfied, the Gauss–Seidel method can allow the simulated body exhibit soft effects. A higher iteration count means the body is more rigid. However, for inextensible effects, it is necessary to ensure that distance constraints are completely fulfilled. Benefitting from the tridiagonal matrix algorithm, the direct solver can be used to ensure the distance constraints are fulfilled in every iteration. The detailed implementation is elaborated in a later section.

The above derivation shows that the dynamic body simulated by the PBD should be initially discretized as a set of positions and orientations. Then, the constraint function should be provided to constrain the state of the positions and orientations. In the solution stage, the main goal is to optimize the solution of the positions and orientations to fulfill the current constraint function. Therefore, the key is to find concise and effective forms of constraint functions to represent dynamic behaviors.

### Algorithm flow of a unified particle framework

One advantage of using PBD to simulate dynamic bodies is that the soft body, rigid body, and elastic rod can be solved with the unified particle framework [19]. In real-time applications of virtual surgery, a functional, whole scene always contains multiple types of dynamic bodies, such as surgical instruments, suture needles, and soft tissues. Integrating the existing PBD and our method to achieve the physical visible for different objects in solving stage greatly improves the stability of the multiple body dynamics. The algorithm flow of the unified particle framework is described below.

**Fig. 1:**
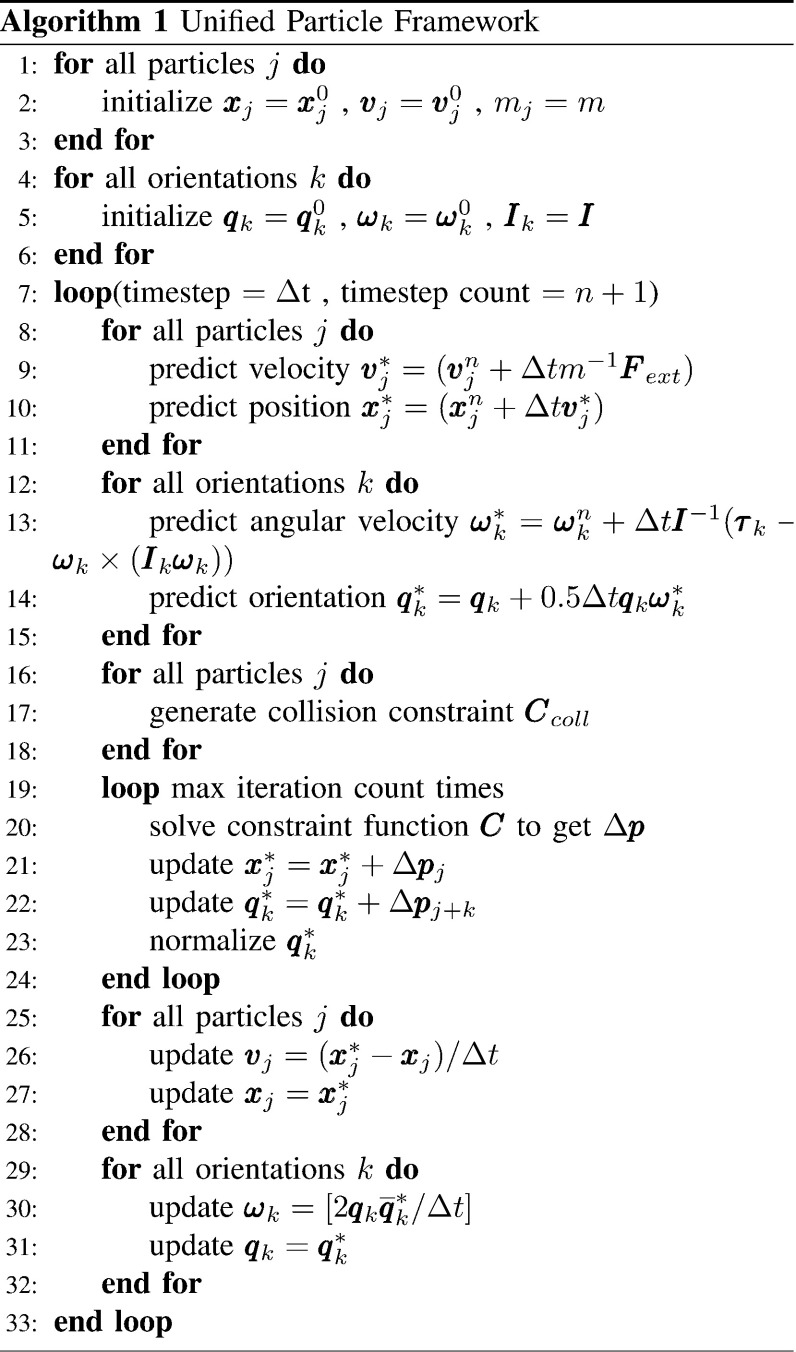
Algorithm flowchart of the unified particle framework

In the algorithm outlined in Fig. 1, positions and orientations are treated in the same way. Before the simulation loop, the position, velocity, and mass of all particles were initialized. Similarly, the quaternion, the angular velocity, and the moment of inertia for all orientations were initialized. To run the simulation step, the explicit Euler integration algorithm was used to predict positions and orientations. In this step, the external force ${{\varvec{F}}}_{{\mathrm{ext}}}$ and torque ${{\varvec{\tau }} }$ are introduced into simulation. In general, gravity is represented as an external force. For simulating surgical thread, the external torque loaded on the thread is set to zero vector. Next, for all collision primitives, continuous collision detection was used to generate a collision constraint based on the state of the previous time step and the current predicted time step. The collision constraints were combined with the general constraint for the next step. Then, the above correction method was used to project the constraints and to identify the appropriate corrections for positions and orientations, which fulfilled the constraint function to determine the correct state of the simulation body. Finally, the linear velocity and angular velocity were updated with the correction positions and orientations.

**Fig. 2:**
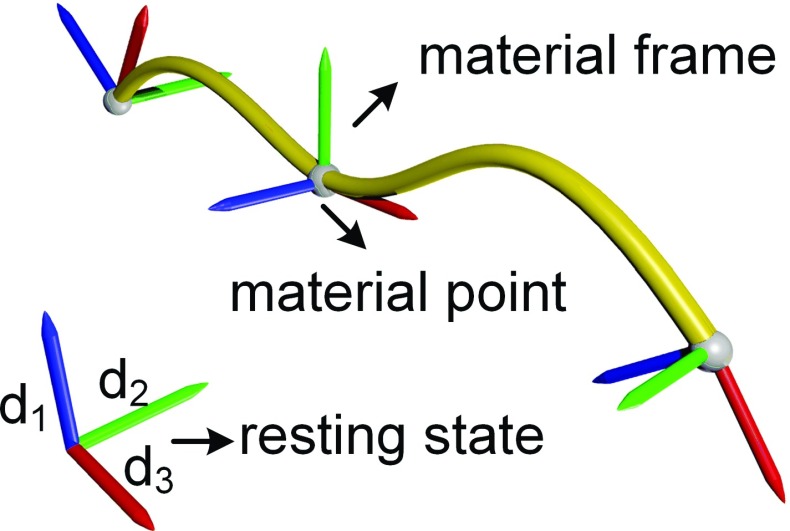
Material frame transformations along the centerline of the elastic rod from start to end

### Discrete Cosserat rod

In the Cosserat rod theory, the centerline of the elastic rod is a directional curve [20]. Using the parameter $s\left[ {0,1} \right]$ to represent the rod from start to end, the vector-valued function ${{\varvec{R}}}\left( s \right)$ represents the material point on the elastic rod. As shown in Fig. 2, to describe the degree of bending and twisting, every material point was attached a material frame, which was modeled with an orthogonal base $\left[ {{{\varvec{d}}}_1 ,{{\varvec{d}}}_2 ,{{\varvec{d}}}_3 } \right]$ in which every vector is referred to as a director. For director ${{\varvec{d}}}_3$, the vector should occur along the same direction as the tangent. For director ${{\varvec{d}}}_2$, the vector should occur along the direction of the principle normal, such that ${{\varvec{d}}}_1 ={{\varvec{d}}}_3 \times {{\varvec{d}}}_2$. Therefore, we can measure the deformation behavior of the elastic rod from the rotation of the material frame along the curve. In differential geometry, the Darboux vector ${ {\varvec{\Omega }} }\left( {s} \right)$ is used to parameterize the frame rotation:

6 $$\begin{aligned} {{\varvec{\Omega }} }\left( {s} \right) =\frac{1}{2} \sum \limits _{k=1}^3 {{\varvec{d}}}_k \left( s \right) \times {{\varvec{d}}}_k \left( s \right) ^{{{\prime }}}, \end{aligned}$$

where the prime (${}^{{\prime }})$ denotes the derivative with respect to s. The projection of the Darboux vector to the material point coordinates was used to measure the bending and twisting strain in the Cosserat rod theory:

7 $$\begin{aligned} {{{\varOmega }} }_i \left( s\right) ={{\varvec{\Omega }} }\left( s \right) \cdot {{\varvec{d}}}_i \left( s \right) \!. \end{aligned}$$

The strain measurement is the difference between the current state (${\varOmega }_1$, ${\varOmega }_2$, ${\varOmega }_3 )$ and the resting configuration state (${\varOmega }_1^0$, ${\varOmega }_2^0$, ${\varOmega }_3^0 )$. ${\varOmega }_1 -{\varOmega }_1^0$ and ${\varOmega }_2 -{\varOmega }_2^0$ parameterize the degree of bending strain, and ${\varOmega }_3 -{\varOmega }_3^0$ parameterizes the degree of twisting strain.

**Fig. 3:**
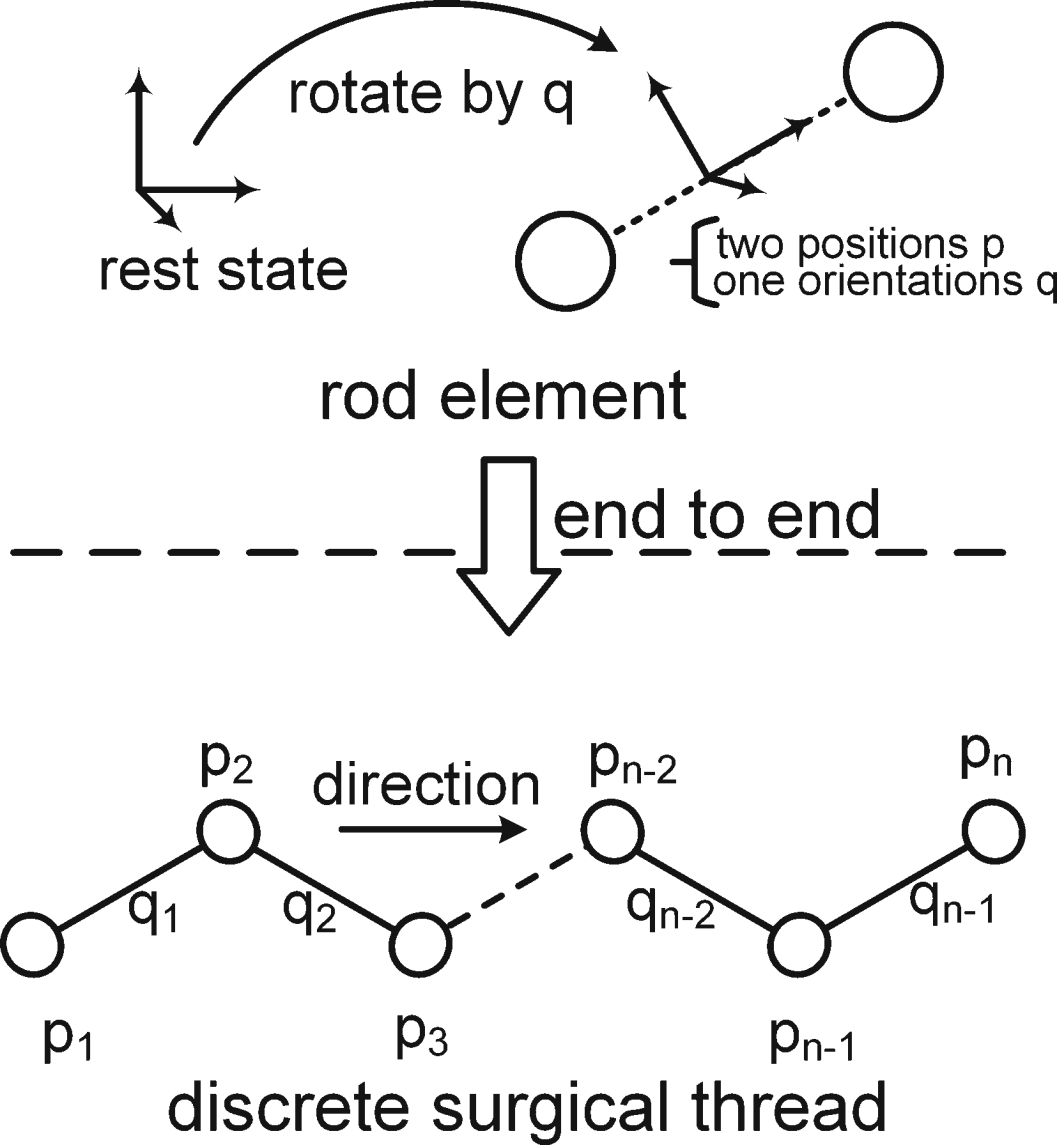
Discrete surgical thread represented by connected rod elements

To introduce the Cosserat rod theory into PBD, the elastic rod should be discrete. Using the theory proposed by Umetani et al. [13] and improved by Kugelstadt and Schömer [15], the curve of the elastic rod centerline is discretized as the rod element. Every rod element is constructed as two particles and one material frame. The rod element is connected by the same particle as the linear structure (Fig. 3). The material frame is parameterized as a quaternion corresponding to the remaining configured material frame. There are two constraint types generated from the Cosserat rod theory [15]. Here, we discuss only some concise clarifications; for more details, please refer to the previous report. First, the elastic rod is inextensible, and therefore, the stretch length should be constrained to the same length as each rod element. Additionally, to correctly describe the bend and twist, the ${{\varvec{d}}}_3$ director should remain parallel to the rod element direction. These two characterizations are integrated into the shear–stretch constraint Eq. (8), in which ${{\varvec{p}}}_1$ and ${{\varvec{p}}}_2$ are the two end particles of the same rod element. ${{\varvec{R}}}\left( {{\varvec{q}}} \right)$ is the rotation matrix constructed by the material frame quaternion. ${{\varvec{e}}}_{3}$ is the resting state of the ${{\varvec{d}}}_{3}$ director:

8 $$\begin{aligned} {{\varvec{C}}}_{s} \left( {{{\varvec{p}}}_{1} ,{{\varvec{p}}}_{2} ,{{\varvec{q}}}} \right) =\frac{1}{l}\left( {{{\varvec{p}}}_2 -{{\varvec{p}}}_1 } \right) -{{\varvec{R}}}\left( {{\varvec{q}}} \right) {{\varvec{e}}}_3 =\mathbf{0}. \end{aligned}$$

Second, for bending and twisting deformation in a continuous rod model, the Darboux vector in the material frame coordinates can be parameterized as the orientation quaternion function:

9 $$\begin{aligned} {\varOmega }=2\bar{{\varvec{q}}}{{{\varvec{q}}}}^{\prime }. \end{aligned}$$

The difference between the resting configurations can be calculated by measuring the strain:

10 $$\begin{aligned} {\varOmega }-{\varOmega }^{0}=2\left( \bar{{\varvec{q}}}{{{\varvec{q}}}}^{\prime }- \bar{{\varvec{q}}}^{0}{{{{\varvec{q}}}}^{\prime }}^{{{\varvec{0}}}}\right) \!. \end{aligned}$$

To solve for bending and twisting strain in PBD, the discrete format of ${\varOmega }-{\varOmega }^{0}$ based on the rod element is derived from the adjacent quaternions through arithmetic mean interpolation. Equation (11) describes the corresponding bend and twist constraint, in which ***q*** and ***u*** are the two adjacent material frame quaternions. The right superscript 0 represents the resting state, and $\mathfrak {I}$ is the imaginary part vector of the quaternion result. In the discrete Cosserat rod, the discrete Darboux vector is parametrized as the imaginary part of a quaternion and used to measure the bending and twisting strain while the material frame rotates along the centerline:

11 $$\begin{aligned} {{\varvec{C}}}_{b} \left( {{{\varvec{q}}},{{\varvec{u}}}} \right) =\mathfrak {I}\left( {\bar{{{\varvec{q}}}}{\varvec{u}}-\bar{{{\varvec{q}}}}^{0}{{\varvec{u}}}^{0}} \right) =\mathbf{0}. \end{aligned}$$

With the above two constraints given by Eqs. (8) and (11), we modeled the nonlinear deformation of surgical thread and then solved the equations in the PBD framework. Next, we explain how our method enhanced the constant length constraint of surgical thread.

### Direct distance constraint (DDC)

From the above shear–stretch constraint, the inextensible effects are coupled with the shear strain. Here, we separated the distance constraint to yield strong stretching resistance:

12 $$\begin{aligned} C_i \left( {{{\varvec{p}}}_i ,{{\varvec{p}}}_{i+1} } \right) =\Vert {{\varvec{p}}}_i -{{\varvec{p}}}_{i+1}\Vert -{d}. \end{aligned}$$

**Fig. 4:**
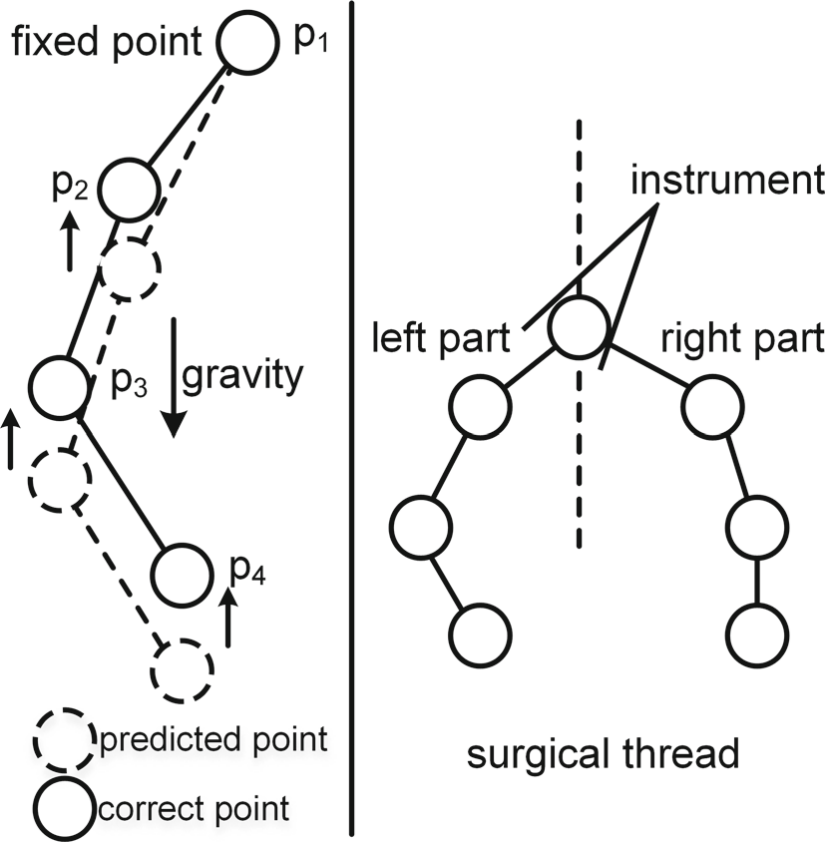
A single surgical thread constrained by two separate direct distance constraints and connected at the instrument holding point

The surgical thread is an elastic rod with a one-dimensional linear structure. Therefore, the distance constraints can be characterized as chains with consecutive particles connected in series. In this situation, the direct solution is an alternative to the iterative solver for projecting the constraint. First, we consider the situation in which the thread is fixed at the start point (Fig. 4). As reported in the work of Han and Harada, by expanding the distance constraint equations, their tridiagonal matrix format can be found and then directly solved [21].

We modified Eq. (3) by changing the format of the equation to yield:

13 $$\begin{aligned} -{{\varvec{C}}}\left( {{\varvec{p}}} \right) =\nabla {{\varvec{C}}}\left( {{\varvec{p}}} \right) \mathbf{W}\nabla {{\varvec{C}}}^{\mathrm{T}}\left( {{\varvec{p}}} \right) {\varvec{\lambda }} , \end{aligned}$$

and we separately considered the distance constraint $C_i \left( {{{\varvec{p}}}_i ,{{\varvec{p}}}_{i+1} } \right)$, which has a gradient of $\frac{\partial C_i }{\partial {\varvec{p}}_i } =-\frac{\partial C_i }{\partial {{\varvec{p}}}_{i+1} }={{\varvec{n}}}_i =\frac{{{\varvec{p}}}_i -{{\varvec{p}}}_{i+1} }{\left| {{{\varvec{p}}}_i -{{\varvec{p}}}_{i+1} } \right| }$. For a distance constraint with only two independent variables, the two corresponding point gradients were nonzero, and the remaining point gradients were zero with the single constraint. Then, we extracted a single column of $C_i \left( {{{\varvec{p}}}_i ,{{\varvec{p}}}_{i+1} } \right)$:

14 $$\begin{aligned} -\,C_i= & {} -\,w_i \frac{\partial C_i }{\partial {\varvec{p}}_i }\frac{\partial C_{i-1} }{\partial {\varvec{p}}_{i-1} }\lambda _{i-1}^{{\prime }} \nonumber \\&+\,\left[ {w_i \left( {\frac{\partial C_i }{\partial {\varvec{p}}_i }} \right) ^{2}+w_{i+1} \left( {-\frac{\partial C_i }{\partial {\varvec{p}}_i }} \right) ^{2}} \right] \lambda _i^{{\prime }}\nonumber \\&-\,w_{i+1} \frac{\partial C_i }{\partial {\varvec{p}}_i }\frac{\partial C_{i+1} }{\partial {\varvec{p}}_{i+1} }\lambda _{i+1}^{{\prime }} . \end{aligned}$$

Initially, the inverse mass of the starting point was fixed at $w_1 =0$, whereas $w_i =1$. Combining the columns and moving the left negative to right side of the equation yielded the new format of Eq. (13):

15 $$\begin{aligned} \left[ {{\begin{array}{c} {C_1 } \\ {C_2 } \\ \vdots \\ {C_{k-1} } \\ {C_k } \\ \end{array} }} \right]= & {} \left[ {{\begin{array}{c@{\quad }c@{\quad }c@{\quad }c@{\quad }c} {-1}&{} {{{\varvec{n}}}_1 {{\varvec{n}}}_2 }&{} 0 &{}\cdots &{} 0 \\ {{{\varvec{n}}}_1 {{\varvec{n}}}_2 }&{} {-2}&{} {{{\varvec{n}}}_2 {{\varvec{n}}}_3 } \cdots &{} 0 \\ \vdots &{} \cdots &{} \ddots &{}\cdots &{}\vdots \\ 0 &{}\cdots &{}{{{\varvec{n}}}_{k-1} {{\varvec{n}}}_{k-2} } &{} {-2} &{}{{{\varvec{n}}}_k {{\varvec{n}}}_{k-1} } \\ 0 &{} \cdots &{}0&{}{{{\varvec{n}}}_k {{\varvec{n}}}_{k-1} } &{}{-2}\\ \end{array} }} \right] \nonumber \\&\times \left[ {{\begin{array}{c} {\lambda _1 } \\ {\lambda _2 } \\ \vdots \\ {\lambda _{{{k}}-1} } \\ {\lambda _{{k}} } \\ \end{array} }} \right] . \end{aligned}$$

The equations make up a typical tridiagonal matrix. The Thomas algorithm was used to directly solve ${\varvec{\lambda }}$ in a single iteration [21]. Then, the correction of the particles can be easily achieved by Eq. (2). If we loop the direct solution into every iteration, the inextensible effect can be immediately achieved.

In most real clinical situations, surgical thread interacts with an instrument. In considering this factor (Fig. 4), a point was fixed to the instrument handle with the inverse of the point mass set to 0. Thus, the surgical thread was implicitly separated into two inextensible elastic rods.

### Continuous collision detection and responses

**Fig. 5:**
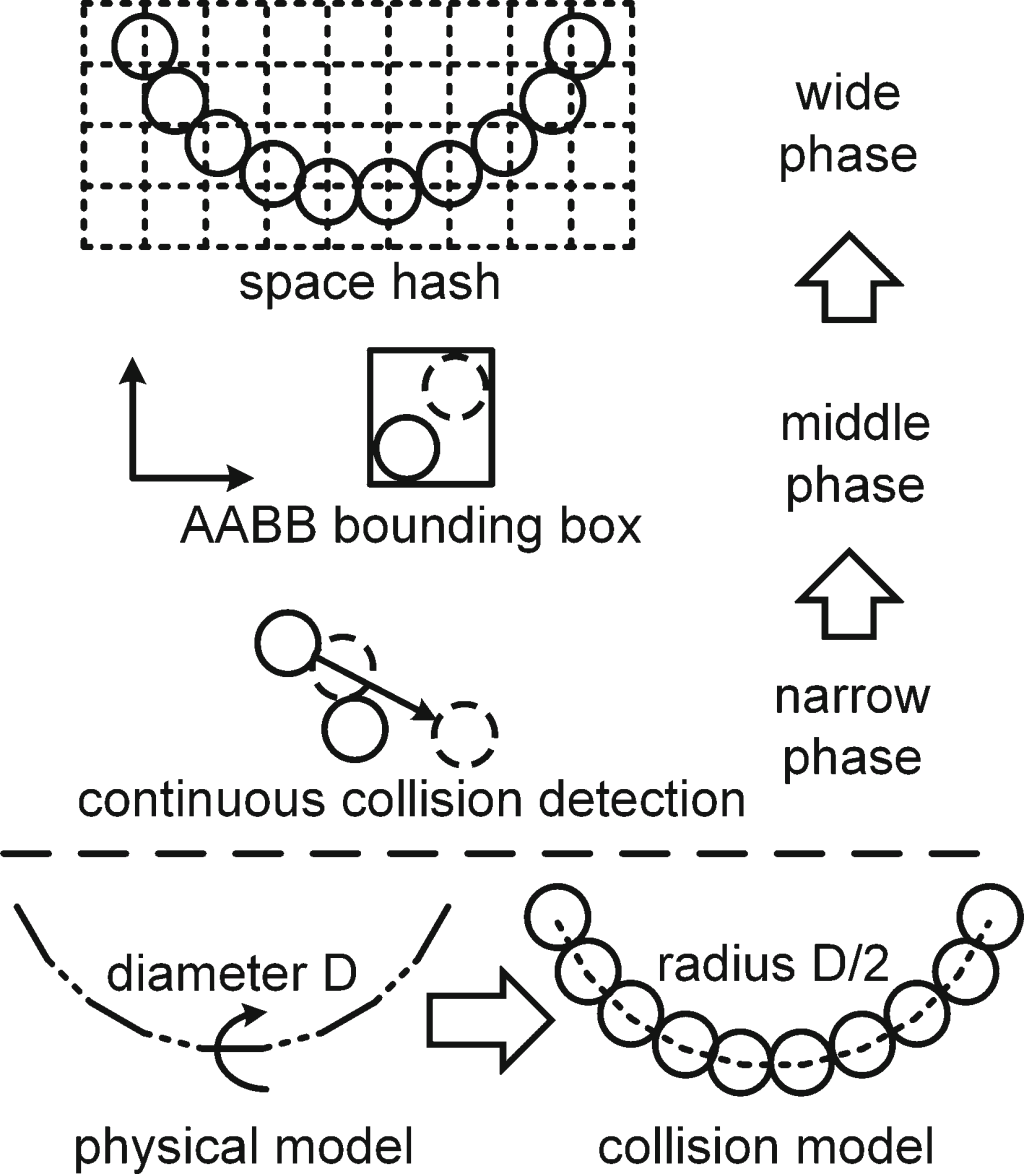
The surgical thread is discretized as closely connected primitive spheres. In the narrow phase, continuous collision detection is used to detect the time of collision. In the middle phase, the AABB bounding box is used to bound the current and predicted positions. In the wide phase, the space hash method is used for culling

In the surgical thread suture process, multiple contacts between threads and instruments occur during knot tying [22]. Due to the large deformation, self-collision and intersection occur very often. Additionally, the diameter of the surgical thread is small, and discrete collision detection would fail under a large relative velocity and simulation time step [23]. Considering the above situation, continuous collision detection based on the spatial hash method was applied to yield robust contact information [24]. Here, we provide a bottom-up interpretation (Fig. 5).

The surgical thread is discretized as fixed-length rod segments that closely connect with each other. Benefiting from the unified size of the primitive segments, the spatial hash method works in appropriate situations. In the existing rope collision detection framework of the narrow phase, the intersection of two lines with no-thickness is generally calculated while they are coplanar. In this case, a cubic equation must be solved. Additionally, if the rope thickness is considered, the extra optimization should be added to avoid the false negative case, which would increase the computational cost. If the entire thread is discretized as closely arranged spheres, only a quadratic equation must be solved in the sphere–sphere case, where the rope thickness is naturally considered to be the sphere radius. Therefore, we chose the thickness of the surgical thread as the rod element length to model the surgical thread. At every nodal point, the collision sphere was generated by the thickness radius (Fig. 5). For continuous sphere–sphere collision detection, the predicted position was used to calculate the route of sphere movement. In the middle phase, the AABB bounding box was used to cover the moving segment of the sphere with the radius offset. In the wide phase, the simulation space was separated as an independent cubic cell. The bounding boxes were hashed to distribute each cell for culling. During the collision detection process, only primitive pairs in the same cell should be tested. The advantage of the above method is that it can support robust, large time step surgical thread simulations. In addition, the space hash method in the wide phase can be easily paralleled on a CPU or a GPU.

**Fig. 6:**
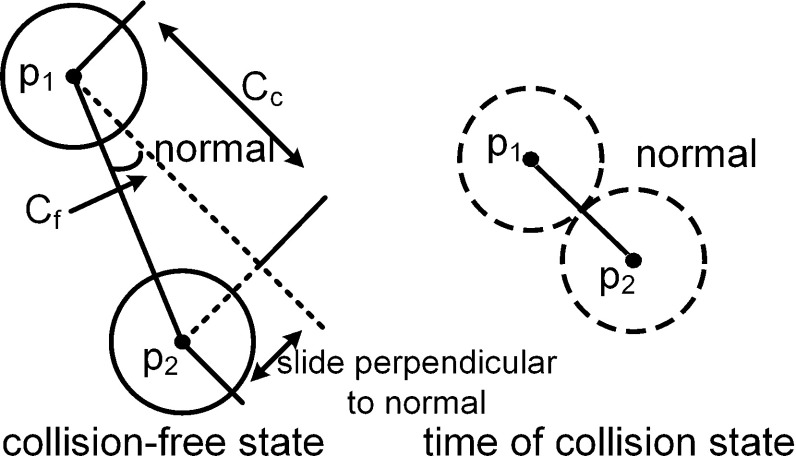
The normal direction is calculated at the time of the collision. Then, the collision response is solved to obtain the collision-free state. $C_{\mathrm{f}}$ measures the direction perpendicular to the normal direction. $C_{\mathrm{c}}$ measures the projection length between the two particles along the normal direction

To apply contact information to increase the simulation stability, the collision response with frictional forces was solved. In most physical simulation methods, the collision response is based on the impulse of velocity or force, whereas solving for friction involves decreasing the velocity. However, in the PBD method, the position of the particles is constrained first, and the velocity is updated later. Therefore, we applied the position-based constraint to handle the collision response and friction to achieve more stable contact resolution. As shown in Fig. 6, there are two collision constraints between two contact particles ${{\varvec{p}}}_1$ and ${{\varvec{p}}}_2$:

- Continuous collision constraint: $C_{\mathrm{c}} \left( {{{\varvec{p}}}_1 ,{{\varvec{p}}}_2 } \right) =\left( {{{\varvec{p}}}_1 -{{\varvec{p}}}_2 } \right) \cdot \mathbf{normal}-{d}$. This constraint maintains the relative position of the two particles as a function of collision time. The distance between two contact particles is larger than the surgical thread diameter to avoid overlap.
- Friction constraint: $C_{\mathrm{f}} \left( {{{\varvec{p}}}_1 ,{{\varvec{p}}}_2 } \right) ={k}\left| {\frac{{{\varvec{p}}}_1 -{{\varvec{p}}}_2 }{\left| {{{\varvec{p}}}_1 -{{\varvec{p}}}_2 } \right| }\times \mathbf{normal}} \right|$ (the gradient derivation in “Appendix” section). This constraint tries to maintain the direction of the current location to match the normal direction in the state of collision time. The parameter *k* is the friction strength and is between 0 and 1; the friction force is smaller when *k* is closer to 0. When $k = 1$, the contact point cannot move perpendicular to the normal direction.

### Energy dissipation

Energy dissipation is a typical feature of surgical thread that distinguishes from ideal Cosserat rod. There are two separate parameters to control the dissipation of surgical thread in our method. One is the damping factor of linear velocity $$d_v $$, and it can achieve the energy dissipation caused by stretching and compressing. Another is the damping factor of angular velocity $$d_a $$, and it can achieve the energy dissipation caused by bending and twisting. In every time step, after finishing the constraint projection process, the linear and angular velocity of each particles is updated by corrections of positions and quaternions. Then, Eqs. (16) and (17) are used to damp the velocity for representing the internal dissipation:

16 $$\begin{aligned} {{\varvec{v}}}= & {} \left( {1-d_v } \right) ^{\Delta t}{{\varvec{v}}}, \end{aligned}$$

17 $$\begin{aligned} {\varvec{\omega }}= & {} \left( {1-d_a } \right) ^{\Delta t}{\varvec{\omega }}, \end{aligned}$$

the $\Delta t$ is the simulation time step. Based on the above equations, the damping factors represent the damping ratio per second of the corresponding velocities. These two parameters are time step independent. The value of damping factor is between 0 and 1. Larger the damping factor, the more energy is dissipated and the model stabilized faster.

**Fig. 7:**
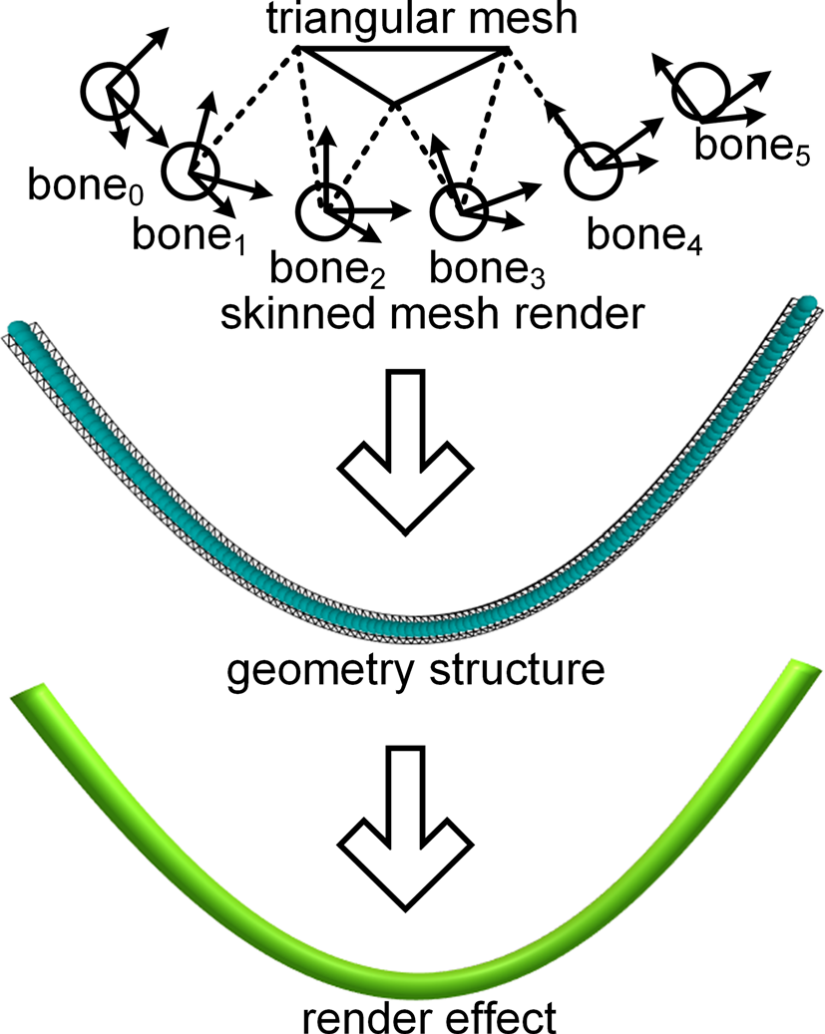
The surgical thread is represented by a surface triangular mesh. The vertex position is determined by the corresponding bone object, in which the weight is inversely proportional to the distance

**Fig. 8:**
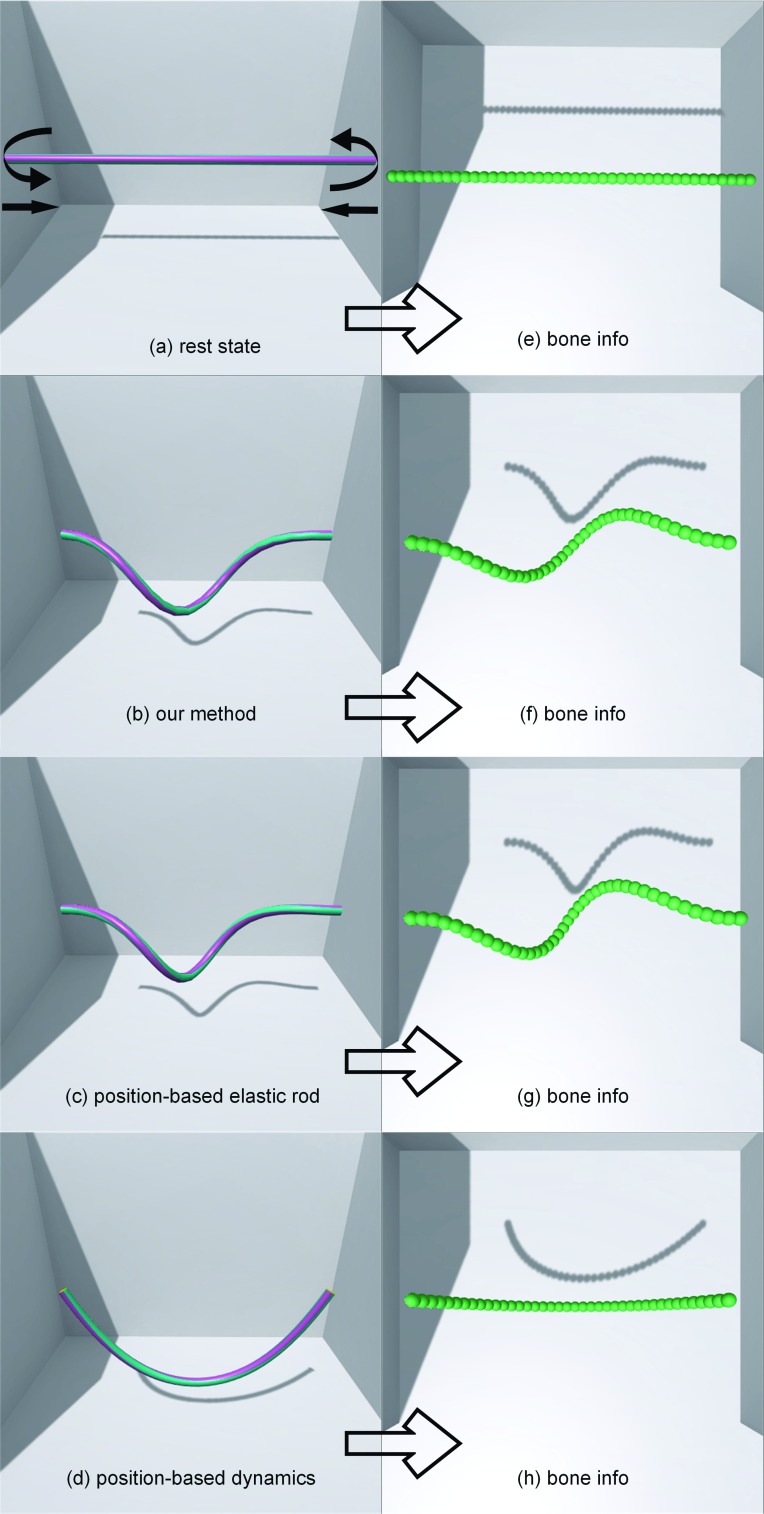
The surgical thread is pinned at the end point as shown in **a** resting state, in which a relative circle rotation and translation bear down on it; **b** method’s bend and twist coupling effect; **c** bend and twist coupling effect in the position-based Cosserat rod; **d** only bend effect in position-based dynamics using distance and edge bending constraints; and **e**–**h** corresponding bone info for the left side from top view

### Model rendering and scene building

The geometrical model to render the scene in real-time is based on triangular mesh data. To render a deformable body, vertex animation technology is used to directly modify the vertex position [25]. Considering that the surgical thread physical model is based on discrete rod elements, a skeletal animation method was used for high-performance rendering. As shown in Fig. 7 and based on geometrical parameters, such as surgical thread length and diameter, triangular mesh data were generated and referred to as skinned mesh. Then, in every particle of the rod element, a bone was defined with the same position and rotation as the rod element and was parameterized as a quaternion. Additionally, for each bone considering the rod element influence area, the bone weight of each vertex was calculated based on the distance of the vertex and bone. In the animation process, the rod element states were first updated from the result of the physical simulation; the bone was then transformed by the rod element; and finally, the vertex position of the mesh data was updated by the bone to achieve the goal of rendering surgical thread. The animation method based on skeletal animation controlled the freedom of the model to simulation nodes and reduced the computational cost for rendering model updates.

**Fig. 9:**
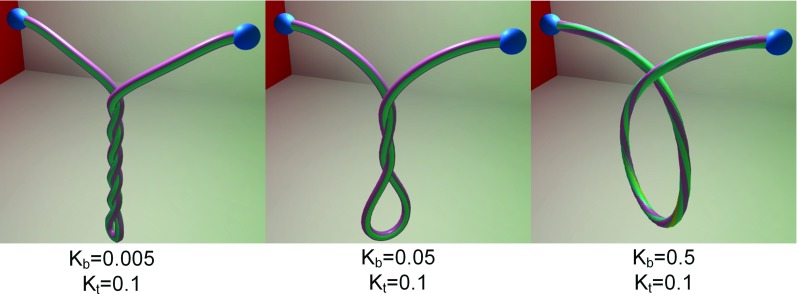
The surgical threads are pinned at the end points with the same twisting constraint stiffness and different bending constraint stiffness. The right end rotates $10\pi$ radians around the world horizontal axis

**Fig. 10:**
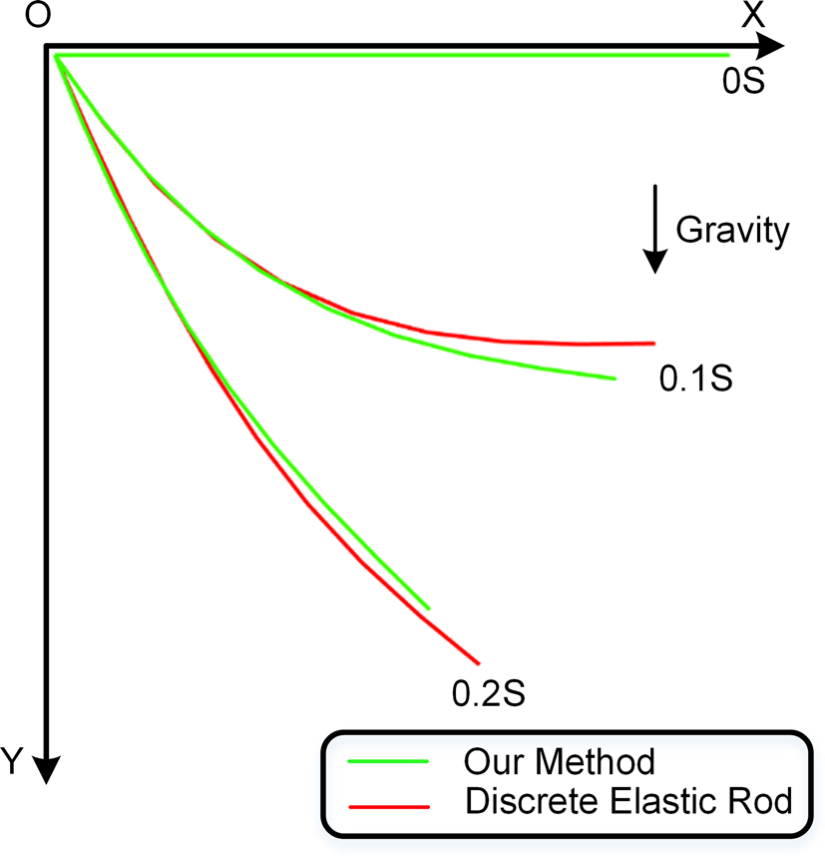
The surgical threads reflects similar visual behavior based on our method and discrete elastic rod. Our method keeps the constant length of the surgical thread

**Fig. 11:**
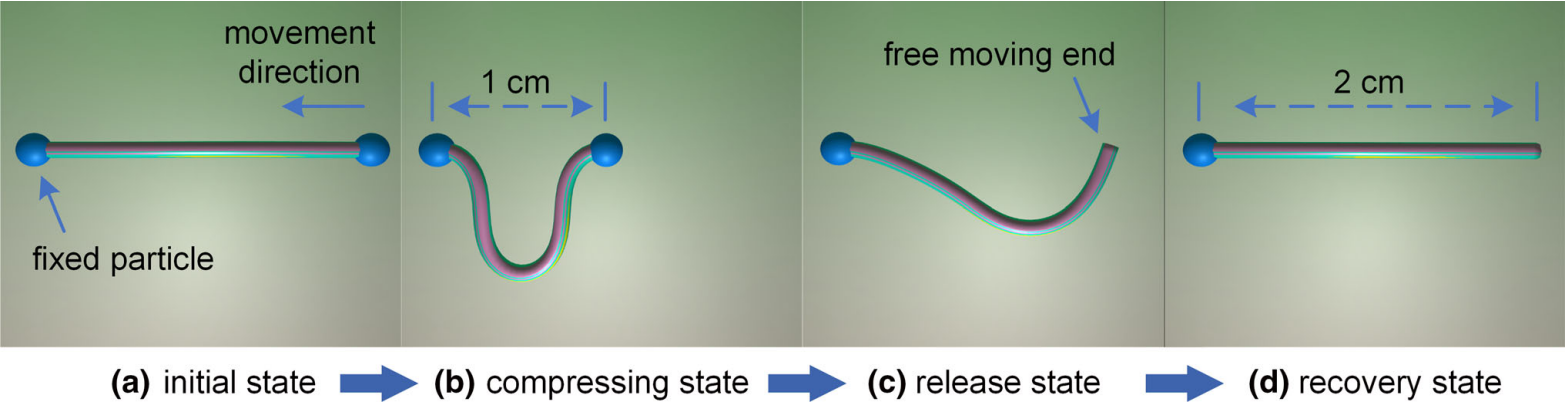
For measuring the controllability of internal dissipation through adjusting damping factor, the surgical thread is compressed and then released

To build a complete high-quality software program, the Unity3D engine was used for multiple platform development [26]. The complex numerical computation and data communication were managed under the cross-platform Mono .NET framework using C# programming language. Thus far, we have explained the implementation of the complete system including calculation and rendering. The above section contains the most vital part of the real-time inextensible surgical thread simulation.

## Results

Our method and test environment were based on the Unity3D engine, which was run on a Windows platform with an Intel Core i7-7700 CPU @ 3.6 GHz and NVIDIA GTX 1070 GPU. The program runs in a single thread without multiple thread optimization for comparing computational costs.

### Comparison between different PBD methods

To test the different PBD method applied to simulate surgical thread, we built a scene with one surgical thread fixed at two ends. The surgical thread was constructed with 40 rod elements. The total length was 15 cm. During the deformation process, the two ends of the surgical thread underwent a 5-cm relative translation with a 360-degree relative rotation. Here, we compare our method with a position-based elastic rods (PBER) proposed by Umetani et al. and PBD that uses distance and bending constraints.

Figure 8 illustrates the final form of the surgical thread using the different methods. We observed that our method and the PBER method generated realistic bending and twisting coupling effects not similar to those of the simple PBD. Additionally, we recorded the physical computation time during the simulation. Our method cost an average of 2.68 ms per frame. The PBER method costs an average of 32.44 ms per frame. The PBD with the simple constraint costs an average of 2.24 ms per frame. Therefore, compared to the PBER method, our method required less time and yielded good frame rate performance with a very similar effect.

Through adjusting the stiffness of bending and twisting constraint, our method is able to simulate surgical threads with different bending and twisting modulus. As shown in Fig. 9, the left end of surgical thread is fixed. The right end rotates $10\pi$ radians around the world horizontal axis. With the constant twisting stiffness ${K}_{\mathrm{t}}$, the surgical thread winds less circles, while the bending stiffness ${K}_{\mathrm{b}}$ decreases. It reflects the positive correlation between constraint stiffness and physical parameters. In addition, a surgical thread is simulated by our method and [9] separately. The left end of surgical thread is fixed, and the right end of it falls down. We record the state of 0, 0.1 and 0.2 s. Figure 10 shows the similar effects of our method to the discrete elastic rod. The bending modulus of discrete elastic rod is 2 GPa, and the twisting modulus is 10 GPa. The bending stiffness of our method is 0.05, and the twisting stiffness of our method is 0.25. However, it is hard to find the exact relationship between the constraints stiffness and the physical parameters. But it is easy to control the model behavior through adjusting the constraint stiffness.

**Fig. 12:**
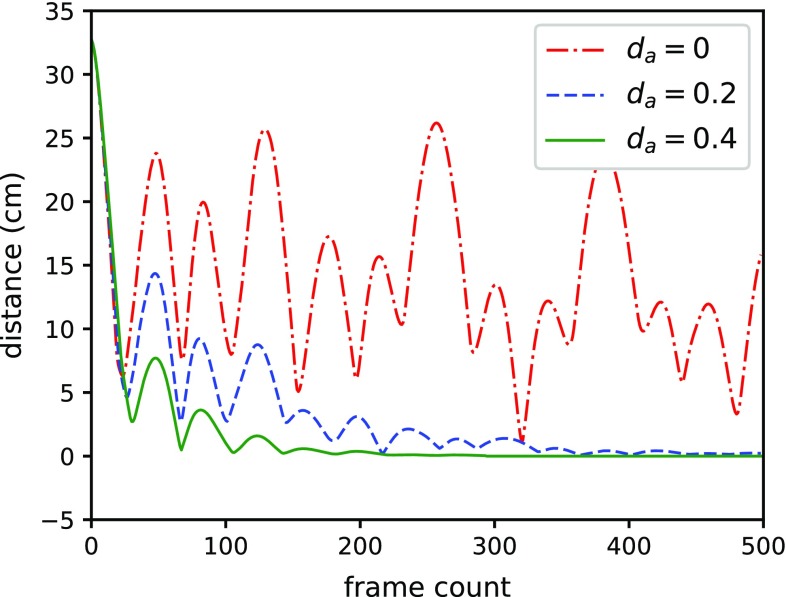
The frame count and total distance relationship under different angular velocity damping factor. The distance is the sum of each particle to their initial state in the corresponding render frame

In order to test the internal dissipation, a surgical thread is simulated with length of 2 cm. There is no friction and gravity force in the environment. The thread is constructed by 49 rod elements. As shown in Fig. 11, the left end of it is fixed, and the right end of it moved 1 cm to compress the thread. Then, the right end is released. The surgical thread reverts to the initial state. We record the surgical thread with angular velocity damping factor $$d_a $$ of 0, 0.2, and 0.4. There are 500 frames captured since the time when right end released. The total distance of each particles to their initial states is calculated as in Fig. 12. When the damping factor is lower, the surgical thread recovery is slower. If the damping factor is 0, the model keeps moving.

**Fig. 13:**
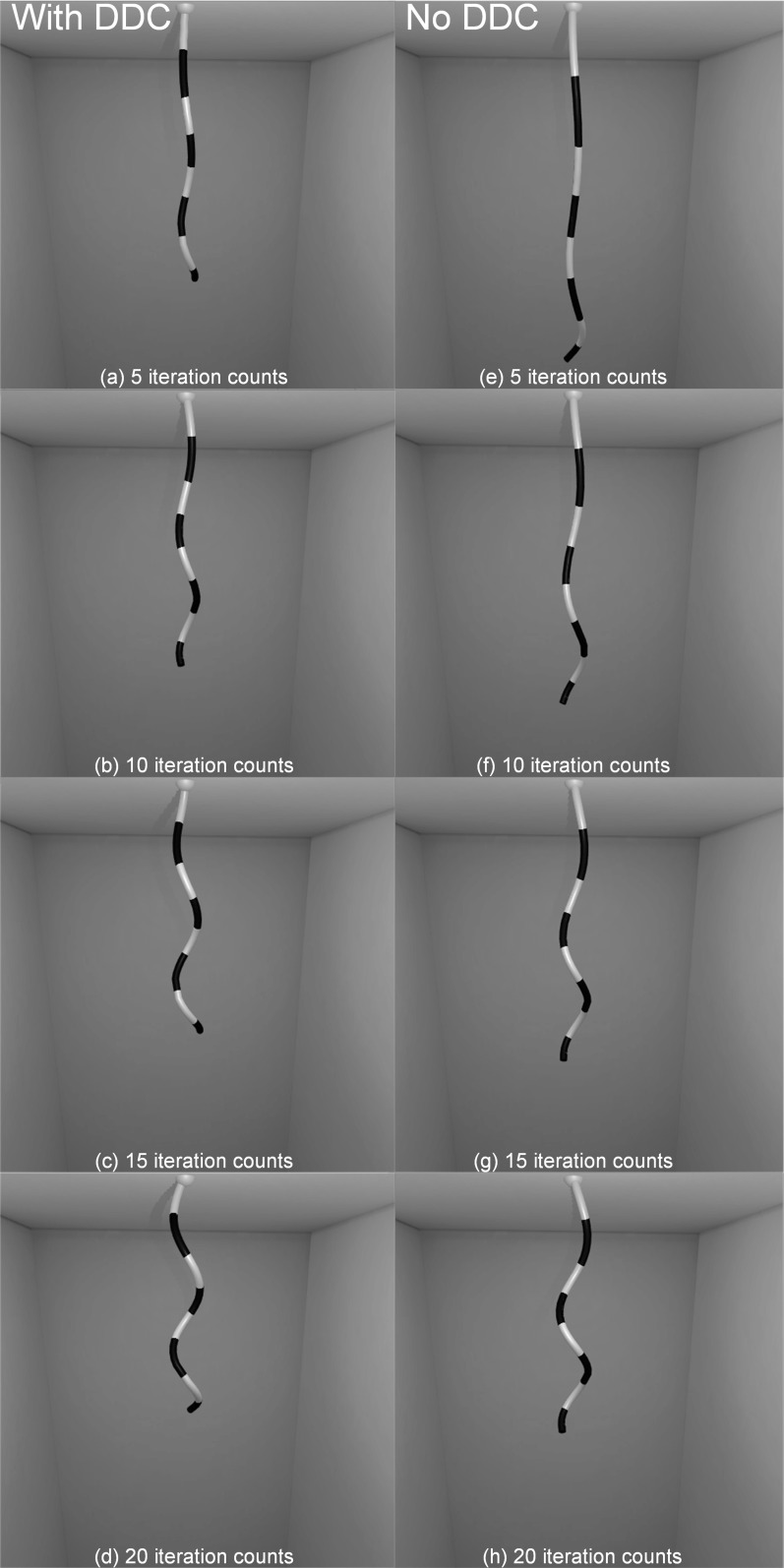
Left side: **a**–**d** surgical thread without the DDC. Right side: **e**–**h** surgical thread with the DDC. The images from top to bottom represent increasing iteration counts from 5 to 40

### Inextensible effect of surgical thread

To test the constraint strength for constant length of the DDC, we compared two surgical threads either with or without the DDC constraints; the other parameters of the surgical thread remained unchanged. The surgical thread is constructed as 120 rod elements, and the total length was 15 cm. In the scene, one end of the surgical thread was pinned, and the other end was dropped and hung freely due to gravity. To illustrate the effect of bending and twisting, the default shape of the surgical thread was a spiral. As shown in Fig. 13, we captured the scene view, the length, and the time cost per frame for 5, 10, 20, and 40 iteration counts.

Intuitively, as shown from left to right in Fig. 13, for the same iteration count, the length of the surgical thread with the DDC was generally smaller than that without the DDC and was closer to the original length.

**Fig. 14:**
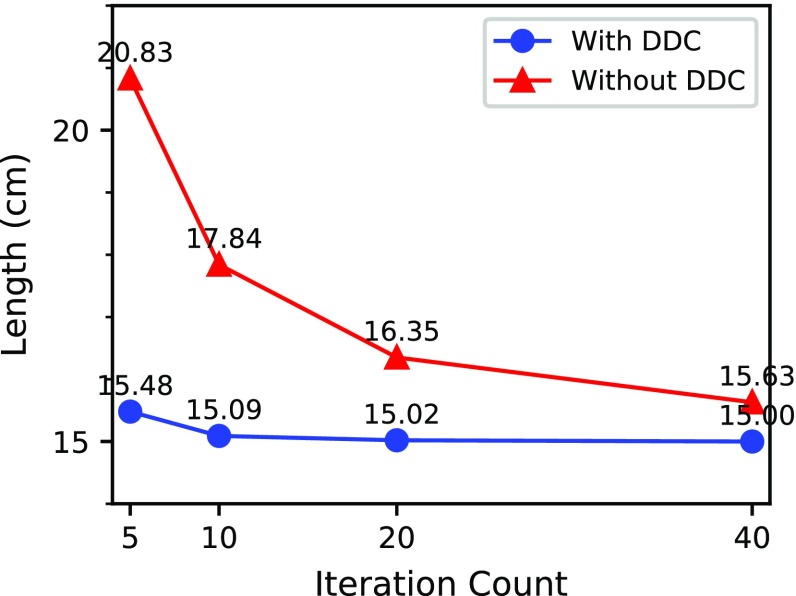
As the iteration count increases, the length becomes more constant. With the DDC, the surgical thread shows smaller elongation

Additionally, as shown by the images in Fig. 13 from top to bottom, for different iteration counts, we observed that the length of the surgical thread without the DDC gradually shortened to the original length. However, the length of surgical thread with the DDC remained stable and was very close to the original length. The above conclusions are depicted more accurately in Fig. 14. When the iteration counts increased, the length elongation of the surgical thread with DDC was always less than 3.2% and remained stable, whereas the length elongation of the surgical thread without the DDC exhibited a decreasing trend. However, the minimum length elongation of 4.2% was achieved at 40 iterations, and the maximum length elongation of 38.9% was observed at 5 iterations, both of which are greater than the elongation with the DDC.

**Fig. 15:**
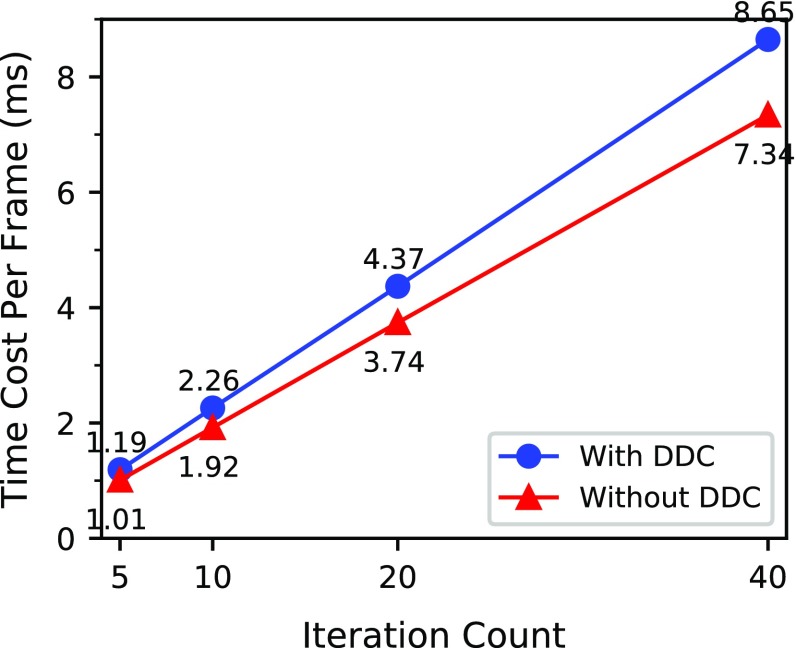
As the iteration count increases, the time cost per frame increases linearly. The introduction of the DDC resulted in a slight increase in the calculation time

Figure 15 presents the time costs of these two surgical thread simulations after different numbers of iterations. For these limited data, the time cost per frame and iteration count were linearly related. The time cost per frame of the surgical thread simulation with the DDC was slightly longer than that without the DDC. However, even after 40 iterations, the time cost remained close to real-time conditions.

The above results show that by introducing the DDC, we achieved an almost constant length of surgical thread, which added a small computational cost. We believe this cost was outweighed by the benefits of improved the simulation stability and subsequent collision detection.

**Fig. 16:**
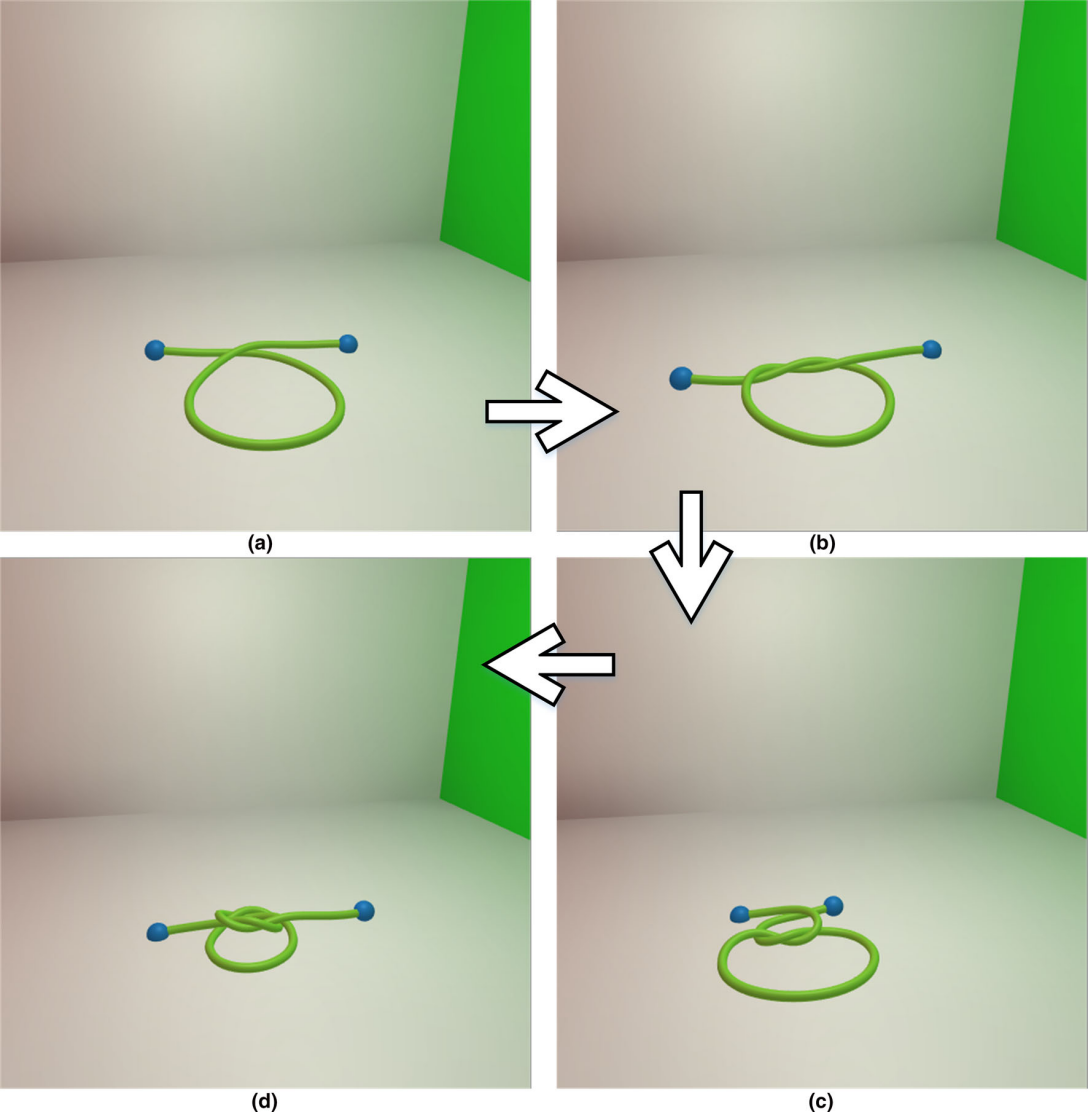
A square knot-tying process is simulated in our method by four steps

### Complex contact resolution

Based on continuous collision detection and collision response constraints, multiple primitive contacts were resolved per iteration loop. This is a significant result for a surgical thread simulation due to the amount of self-collision that occurs during the suturing process. Figure 16 simulates a single surgical thread tied in a square knot, which consists of two throws. In Fig. 16a, the throws are constructed by crossing the ends of the suture to create a loop, one end of which is then wrapped around the other end. In Fig. 16b, one end has been wrapped around the other end by passing it under the long end and up through the loop to complete the first throw. In Fig. 16c, the second throw also begins with one end crossing the other end, which is the same as the first throw. In Fig. 16d, the knot has been tightened, and the result is a square knot.

**Fig. 17:**
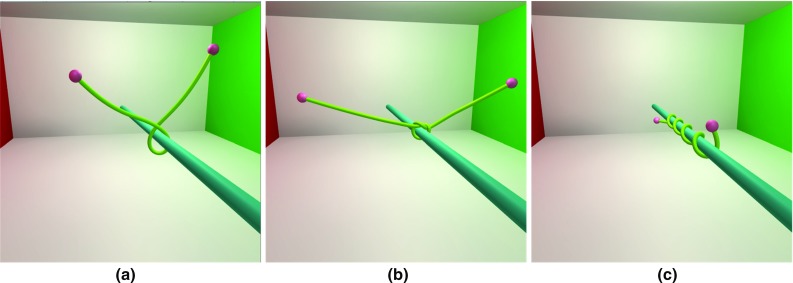
A surgical thread is tied with a rigid body in three steps: **a** resting state, **b** basic knot, **c** wrapping around

**Fig. 18:**
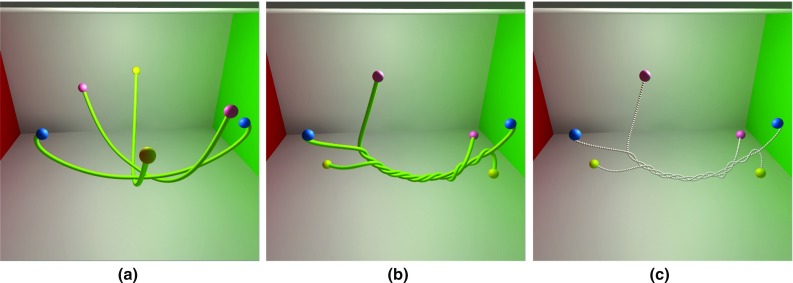
Multiple surgical threads under two complex twining states: **a** resting state, **b** twined state; **c** physical information relating to the thread

To check the interaction between surgical thread and a rigid body, a cylinder was constructed with surgical thread (Fig. 17), and a single throw was tied around the cylinder as shown in Fig. 17b. Figure 17c shows that the friction constraint between the rigid body and the surgical thread caused by spiral winding indicated no relative slip.

We constructed three surgical threads wound around each other as shown in Fig. 18. Almost three complete threads were required for collision constraints, whereas the effect remained stable without sliding or penetrating. Figure 18c illustrates the corresponding solution results of the physical particles.

### Soft tissue suturing simulation

**Fig. 19:**
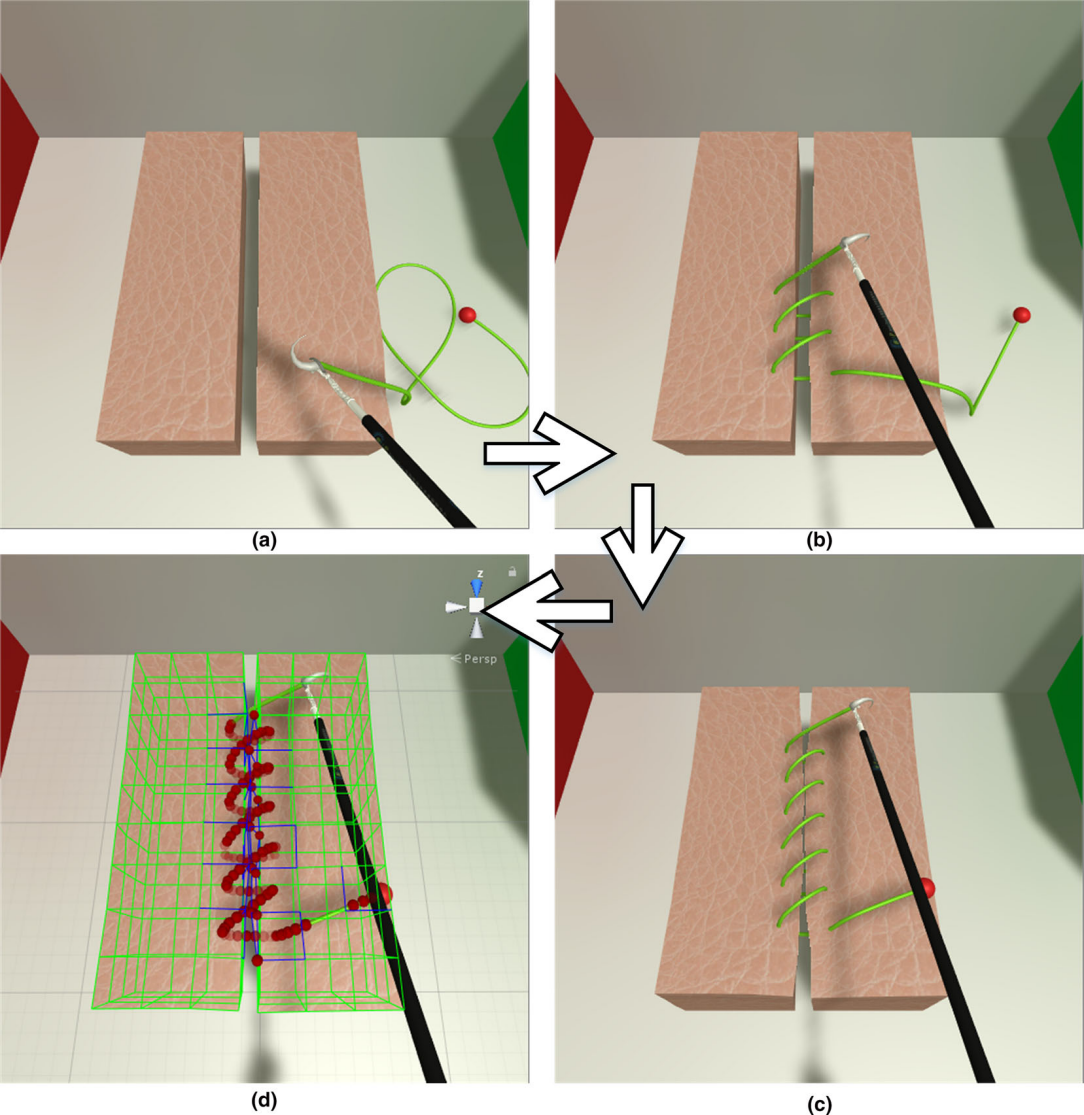
Soft tissue suturing scenes depicting three states: **a** resting state, **b** the surgical thread penetrating the soft tissue, **c** the completed suturing process; **d** corresponding collision information with the thread collision primitive (red dots) and soft tissue collision primitive (blue edge)

Finally, a soft tissue suturing simulation was built based on the unified particle framework. As Fig. 19 shows, the scenes contained the dynamic body of a cubic soft tissue, a suture needle with an instrument handle, and surgical thread. Figure 19a shows the resting state of the interaction between the surgical thread and the soft tissue body; Fig. 19b shows the surgical thread passing through the soft tissue body; Fig. 19c demonstrates the final state of surgical thread suturing in which the two separated soft tissue bodies are pulled together by the surgical thread. The collision information is shown in Fig. 19d, in which the soft tissue was constructed as edge primitives, and the surgical thread was constructed as sphere primitives. The collision pair of the blue-colored edge and the red-colored sphere was used to generate the collision constraint, which was then corrected to the appropriate position for the final rendering. The soft tissue was represented by a soft body. The suture needle and the instrument handle are represented by a rigid body. The surgical thread is represented as an elastic rod. All the dynamic bodies were calculated using the unified particle framework to achieve physical visible interactions in a stable simulation. Due to the unified simulation of multiple dynamics, the entire virtual surgery system can be quickly and accurately built.

## Conclusions

This paper discusses the key methods used for the surgical suturing simulation. Our method based on PBD achieved a unified particle framework to simulate rigid body, elastic rod and soft body. To recreate the inextensible characteristics of surgical thread, the direct solution of the distance constraint was derived from the one-dimensional linear geometrical structure based on the tridiagonal matrix algorithm. This method fulfills the distance constraint per iteration to incorporate a constant length of surgical thread with the shear–stretch constraint. To stabilize the collision response and to solve for the friction in order to simulate complicated knot tying and binding, primitive particle continuous collision detection was used to achieve a large time step and accurate collision detection. Then, the collision response and friction constraint were applied to the constraint projection step for a fast, stable simulation. Finally, by building the soft tissue suturing simulation scene, the interaction between the surgical thread and soft tissue was simulated. The program can run on a single CPU core in real-time without multiple thread optimization.

**Fig. 20:**
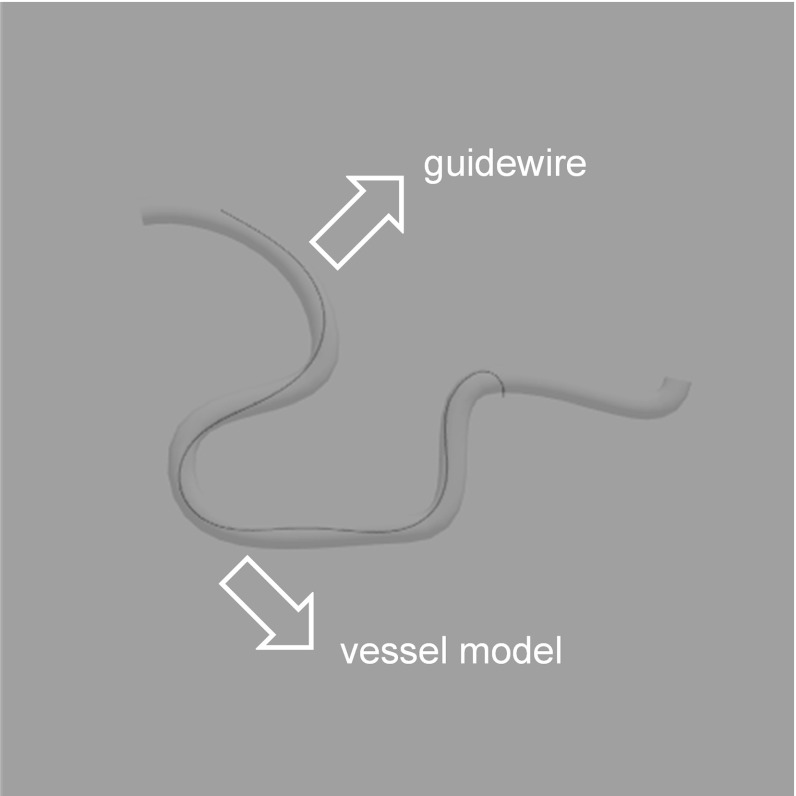
Interventional guidewire demonstration simulated in the unified particle framework

Due to long-standing problems with PBD, simulation effects are related to the iteration count and stiffness parameter. Additionally, the deformation calculation is based on directly correcting the position of particles, and therefore, the force-related effects cannot be easily calculated. In the future, extended position-based dynamics (XPBD) can be used to decouple the iteration count to more easily control simulation effects [27, 28]. For more precisely representing the Coulomb model, the relationship between constraint stiffness and embedding distance should be considered during the collision of two segments.

In virtual surgery system, force feedback can be used to measure the effectiveness of operation and enhance the immersion of operators. Although in our method, it is not able to directly obtain the force on the surgical thread. But still there are two ways to extend it for force-based application. One is trying to integrate exist force-based method with PBD, such as mass spring model or finite element method. Another way is to extend the PBD, such as the XPBD, it introduces the constraint energy and compliance parameter for measuring the constraint force, and it only increases a little time consumption and can be applied to our method directly. Therefore, it is easily to produce the force for later force-based application based on our method. Meanwhile, the mechanics experiment on the real surgical thread should be conducted to measure the accuracy of those simulation methods.

Additionally, considering the general applications of the Cosserat rod to the medical field, the interventional guidewire and the gastric endoscope could also be simulated using our framework. Figure 20 presents a demonstration of the interventional operation process, in which the guidewire reflects real characteristics, including twisting and bending effects. Therefore, it is important to extend the applications of our method.

## Appendix: Friction constraint gradient derivation

The friction constraint is $C_{\mathrm{f}} \left( {{{\varvec{p}}}_{{\varvec{1}}} ,{{\varvec{p}}}_{{\varvec{2}}} } \right) ={k}\left| {\frac{{{\varvec{p}}}_{{\varvec{1}}} -{{\varvec{p}}}_{{\varvec{2}}} }{\left| {{{\varvec{p}}}_{{\varvec{1}}} -{{\varvec{p}}}_{{\varvec{2}}} } \right| }\times \mathbf{normal}} \right|$. We set ${{\varvec{q}}}=\frac{{{\varvec{p}}}_{{\varvec{1}}} -{{\varvec{p}}}_{{\varvec{2}}} }{\left| {{{\varvec{p}}}_{{\varvec{1}}} -{{\varvec{p}}}_{{\varvec{2}}} } \right| }\times \mathbf{normal}$. The derivative with respect to ${{\varvec{p}}}_{{\varvec{1}}}$ is

18 $$\begin{aligned} \frac{\partial C_{\mathrm{f}} \left( {{{\varvec{p}}}_{{\varvec{1}}} ,{{\varvec{p}}}_{{\varvec{2}}} } \right) }{\partial {{\varvec{p}}}_{{\varvec{1}}} }=\left( {\frac{\partial {{\varvec{q}}}}{\partial {{\varvec{p}}}_{{\varvec{1}}} }} \right) ^{\mathrm{T}}\frac{\partial \left( {{k}\left| {{\varvec{q}}} \right| } \right) }{\partial {{\varvec{q}}}}. \end{aligned}$$

We calculate the left and right part of Eq. (18) separately. The right part is

19 $$\begin{aligned} \frac{\partial \left( {{k}\left| {{\varvec{q}}} \right| } \right) }{\partial {{\varvec{q}}}}={k}\frac{{{\varvec{q}}}}{\left| {{\varvec{q}}} \right| }={k}\frac{\frac{{{\varvec{p}}}_{{\varvec{1}}} -{{\varvec{p}}}_{{\varvec{2}}} }{\left| {{{\varvec{p}}}_{{\varvec{1}}} -{{\varvec{p}}}_{{\varvec{2}}} } \right| }\times \mathbf{normal}}{\left| {\frac{{{\varvec{p}}}_{{\varvec{1}}} -{{\varvec{p}}}_{{\varvec{2}}} }{\left| {{{\varvec{p}}}_{{\varvec{1}}} -{{\varvec{p}}}_{{\varvec{2}}} } \right| }\times \mathbf{normal}} \right| }. \end{aligned}$$

The left part in the transpose is

20 $$\begin{aligned} \frac{\partial {{\varvec{q}}}}{\partial {{\varvec{p}}}_{{\varvec{1}}} }=\frac{\partial \left( {\frac{{{\varvec{p}}}_{{\varvec{1}}} -{{\varvec{p}}}_{{\varvec{2}}} }{\left| {{{\varvec{p}}}_{{\varvec{1}}} -{{\varvec{p}}}_{{\varvec{2}}} } \right| }\times \mathbf{normal}} \right) }{\partial {{\varvec{p}}}_{{\varvec{1}}} }. \end{aligned}$$

The cross product matrix is used to convert the cross product to matrix multiplication:

21 $$\begin{aligned}&\frac{\partial \left( {\frac{{{\varvec{p}}}_{{\varvec{1}}} -{{\varvec{p}}}_{{\varvec{2}}} }{\left| {{{\varvec{p}}}_{{\varvec{1}}} -{{\varvec{p}}}_{{\varvec{2}}} } \right| }\times \mathbf{normal}} \right) }{\partial {\varvec{p}}_{{\varvec{1}}} }=\frac{\partial \left( {\left[ {-\mathbf{normal}} \right] \times \frac{{\varvec{p}}_{{\varvec{1}}} -{\varvec{p}}_{{\varvec{2}}} }{\left| {{\varvec{p}}_{{\varvec{1}}} -{\varvec{p}}_{{\varvec{2}}} } \right| }} \right) }{\partial {\varvec{p}}_{{\varvec{1}}} }\nonumber \\&\quad =\left[ {-\mathbf{normal}} \right] \times \frac{\partial \left( {\frac{{\varvec{p}}_{{\varvec{1}}} -{\varvec{p}}_{{\varvec{2}}} }{\left| {{\varvec{p}}_{{\varvec{1}}} -{\varvec{p}}_{{\varvec{2}}} } \right| }} \right) }{\partial {\varvec{p}}_{{\varvec{1}}} }, \end{aligned}$$

22 $$\begin{aligned}&=\left[ {-\mathbf{normal}} \right] \times \frac{\mathbf{I}\left| {{\varvec{p}}_{{\varvec{1}}} -{\varvec{p}}_{{\varvec{2}}} } \right| ^{2}-\left( {{\varvec{p}}_{{\varvec{1}}} -{\varvec{p}}_{{\varvec{2}}} } \right) \left( {{\varvec{p}}_{{\varvec{1}}} -{\varvec{p}}_{{\varvec{2}}} } \right) ^{\mathrm{T}}}{\left| {{\varvec{p}}_{{\varvec{1}}} -{\varvec{p}}_{{\varvec{2}}} } \right| ^{3}}.\nonumber \\ \end{aligned}$$

Substituting Eqs. (19) and (22) into Eq. (18), we get

23 $$\begin{aligned} \frac{\partial C_{\mathrm{f}} \left( {{\varvec{p}}_{{\varvec{1}}} ,{\varvec{p}}_{{\varvec{2}}} } \right) }{\partial {\varvec{p}}_{{\varvec{1}}} }= & {} \left( {\left[ {-\mathbf{normal}} \right] \times \frac{I\left| {{\varvec{p}}_{{\varvec{1}}} -{\varvec{p}}_{{\varvec{2}}} } \right| ^{2}-\left( {{\varvec{p}}_{{\varvec{1}}} -{\varvec{p}}_{{\varvec{2}}} } \right) \left( {{\varvec{p}}_{{\varvec{1}}} -{\varvec{p}}_{{\varvec{2}}} } \right) ^{\mathrm{T}}}{\left| {{\varvec{p}}_{{\varvec{1}}} -{\varvec{p}}_{{\varvec{2}}} } \right| ^{3}}} \right) {T}_{{k}}\nonumber \\&\times \frac{\frac{{\varvec{p}}_{{\varvec{1}}} -{\varvec{p}}_{{\varvec{2}}} }{\left| {{\varvec{p}}_{{\varvec{1}}} -{\varvec{p}}_{{\varvec{2}}} } \right| }\times \mathbf{normal}}{\left| {\frac{{\varvec{p}}_{{\varvec{1}}} -{\varvec{p}}_{{\varvec{2}}} }{\left| {{\varvec{p}}_{{\varvec{1}}} -{\varvec{p}}_{{\varvec{2}}} } \right| }\times \mathbf{normal}} \right| }. \end{aligned}$$

And the derivative with respect to ${\varvec{p}}_{{\varvec{2}}}$ is

24 $$\begin{aligned} \frac{\partial C_{\mathrm{f}} \left( {{\varvec{p}}_{{\varvec{1}}} ,{\varvec{p}}_{{\varvec{2}}} } \right) }{\partial {\varvec{p}}_{{\varvec{2}}} }=-\frac{\partial C_{\mathrm{f}} \left( {{\varvec{p}}_{{\varvec{1}}} ,{\varvec{p}}_{{\varvec{2}}} } \right) }{\partial {\varvec{p}}_{{\varvec{1}}} }. \end{aligned}$$

**Rule 1** For scalar *y*, column vector $\mathbf{u}$ and $\mathbf{x}$, if $y=f\left( \mathbf{u} \right) ,\mathbf{u}={g}\left( \mathbf{x} \right)$, we can get $\frac{\partial f}{\partial \mathbf{x}}=\left( {\frac{\partial \mathbf{u}}{\partial \mathbf{x}}} \right) ^{\mathrm{T}}\frac{\partial f}{\partial \mathbf{u}}$.

**Rule 2** For vector **a** and vector **b**, $\mathbf{a}\times \mathbf{b}=-\mathbf{b}\times \mathbf{a}$.

**Rule 3** For vector **a** and vector **b**, the cross product can be expressed as matrix multiplication:

25 $$\begin{aligned} \mathbf{a}\times \mathbf{b}=\left[ \mathbf{a} \right] \times \mathbf{b}=\left[ {{\begin{array}{l@{\quad }l@{\quad }l} 0&{} {-a_3 }&{} {a_2 } \\ {a_3 }&{} 0&{} {-a_1 } \\ {-a_2 }&{} {a_1 }&{} 0 \\ \end{array} }} \right] \left[ {{\begin{array}{l} {b_1 } \\ {b_2 } \\ {b_3 } \\ \end{array} }} \right] . \end{aligned}$$
